# Metabolic Profile of Breast Cancer in a Population of Women in Southern Spain

**DOI:** 10.2174/1874189400802010001

**Published:** 2008-01-18

**Authors:** Juan-Bosco Lopez-Saez, Jose Antonio Martinez-Rubio, Maria Montes Alvarez, Carmen Gonzalez Carrera, Margarita Dominguez Villar, Antonio Garcia de Lomas Mier, Charo Doménech, Avelino Senra-Varela

**Affiliations:** Departamento de Medicina, Facultad de Medicina, Universidad de Cádiz, Cádiz, Spain

**Keywords:** Case-control study, breast cancer, carcinogenesis, metabolic profile.

## Abstract

**Background:** There are indications that mortality in breast cancer is related with dietary factors, but no study has been large enough to characterise reliably how, this risk is influenced. To establish a logistic regression equation that would predict breast cancer from factors in the endocrinological and metabolic profile, we studied endocrinological and metabolic risk factors that are modified by the diet, in a population of women with breast cancer in southern Spain.

**Patients and Methods:** We carried out a simple a case-control study comparing 204 women with breast cancer (96 premenopausal and 108 postmenopausal women) and 250 healthy control subjects. The predictive variables were basal glycaemia, insulin, glycosylated haemoglobin (HbA1c), C-peptide, insulin-like growth factor-I (IGF-I), total cholesterol, triglycerides, high density lipoprotein-c (HDL-C), low density lipoprotein-c (LDL-C), selenium and Quetelet index (BMI).

**Results:** The metabolic profile differed between pre- and postmenopausal patients, and metabolic alterations were greater in postmenopausal than in premenopausal women. The differences between healthy subjects and breast cancer patients were clearly significant.

**Conclusions:** Our findings have several potential practical applications in the early detection of breast cancer, especially in premenopausal women; in primary prevention; and in the development of a mathematical model of breast carcinogenesis.

## INTRODUCTION

Breast carcinoma includes a heterogeneous group of tumors with variable prognosis. International rates of breast cancer between countries suggests that environmental factors, perhaps dietary, may influence the risk of this disease. We investigated some endocrine and metabolic factors influenced by diet to establish profile of breast cancer. In Spain, incidence of breast cancer increased is 2.26 times the mortality from this tumour [1] and mortality from breast cancer increased 122% in absolute terms (62% adjusted rate) from 1961 to 1980 [2]. It are look at the changes in Spain during this period in comparison with the preceding years it shows that socioeconomic level increased significantly, and so did food availability and consumption [3]. During the same period there was a clear decrease in mortality from stomach cancer [4]. Decreasing mortality by gastric cancer showed a significant inverse correlation with mortality from breast cancer and quantitative variations in the diet with time, and differences in the diet between regions; in fact, mortality from breast cancer was significantly greater in coastal than in inland provinces and the contrary was happening with stomach cancer: the link between both is diet [4–6].

We have found that although menarche occurs one year later in northern than in southern Spain (because of the geographical influence of latitude), mortality from breast cancer is much higher in the former than in the latter part of the country [6]. Women who became obese as adults, the degree of obesity was inversely related with age at menarche: the greater the Quetelet index in adulthood, the younger the age at menarche [7]. This finding suggests that early menarche is not a primary risk factor for breast cancer, but is rather a consequence of the increasing socioeconomic level in Spain and the diet [3]. The epidemiological evidence of an association between dietary fat and the development of breast cancer is strong and is supported by numerous animal models. Several studies have identified an association between increasing body mass index and advanced stage breast cancer [8].

Postmenopausal women with breast cancer were found to have a significant degree of relative hyperglycaemia; although basal glycaemia was within the normal range, the values were higher than in normal control subjects [8]. Moreover, insulin levels and C-peptide values [9] correlated directly with basal glycaemia [10]. Blood glucose concentrations vary widely through a 24 hours period, and HbA1c is used as a more accurate measure of these daily variations [11]. According to Jackson *et al.* insulin-like growth factor I (IGF-I) have clearly defined mitogenic effect on mammary cancer cells and it is possible that they promote carcinogene-sis in human mammary tissue, protecting cells from apopto-sis and enhance metastasis [12]. Hankinson *et al.* reported that high circulation IGF-I concentrations would be associated with an increased risk of breast cancer patients [13].

Electrocardiography studies of the prevalence of cardiac insufficiency in 512 women with breast cancer revealed a significant increase in signs of coronary insufficiency in these patients in comparison with healthy control subjects, these observations suggest, a possible relation between coronary atherosclerosis and breast cancer [14].

Angiotensin-converting enzyme (ACE) activity was also investigated in patients with breast cancer and was elevated in women in complete remission in both cases comparing the values with normal controls [15].

Zielinski *et al.* found a relative increase in serum total cholesterol in patients with breast cancer [16]. HDL-C levels are also influenced by diet. Because this factor has been little studied in breast cancer patients [17]. We searched for possible relationships between HDL-C levels and breast cancer.

The relation between selenium and the etiology of cancer in humans remains elusive and intriguing, despite the number of epidemiologic studies published on the topic. Low levels of selenium have been associated with a higher risk of cardiovascular diseases and cancer in humans, which is another important factor related to dietary intake [18]. This element has been shown to be decreased in women with breast cancer [19]. The effects of the insulin as a cellular growth factor is partly mediated by the IGF-I [20]. The International Agency for Research on Cancer (IARC) and other cancer research organisations periodically publish list of human carcinogens [21].

In this article, we analyse some arguments that support the important effect of diet on breast carcinogenesis and metabolic and endocrine parameters that are modified by diet.

The aim of the present study was therefore to develop a logistic regression equation that would predict a diagnosis of breast cancer on the basis of metabolic parameters, selecting a combinations of variables with the best sensitivity and specificity. Evidence from population-based studies might suggest reliable biomarkers of breast cancer risk among conventional clinical variables, that could provide a strategy for identifying high-risk individuals who might benefit from preventive intervention.

## PATIENTS AND METHODS

### Study Design

A simple case-control study reported here was designed to search for a possible relation between endocrine and metabolic parameters and the likelihood of breast cancer. We compared the values for these parameters in patients and healthy controls in pre- and postmenopausal women. Variables that differed significantly between patients and controls were included in a mathematical model used to calculate the probability of a diagnosis of breast cancer.

### Participants

We identified 215 women who had been diagnosed with breast cancer admitted to any of the hospitals in which such patients were treated. Of the original sample, 204 were eligible cases (96 pre- and 108 postmenopausal patients). The mean age was 52 years (range: 32-80). Reasons for exclusion being: diagnosis not confirmed by histopathology [6], advanced stage at time of diagnosis [3], or patient’s refusal [2]. We included women who had been operated on recently, women whose disease was in prolonged clinical remission, and women with just detected active recurrence that had not yet been treated with chemotherapy/hormotherapy/inmmu-notherapy/radiotherapy. All the cases were ductal infiltrating carcinoma, classified according to the World Health Organisation classification of breast tumours (WHO) [22].

Control subjects, ascertained in the same time period, were randomised of the population residing in each of the areas. 250 apparently healthy women (with no clinical or mammography evidence of malignancy in the breast), of the same age group and share the same environmental were selected. All cases with advanced or recently treated disease or carriers of the other chronic disease, included arterial hypertension, psychiatric disease, congestive heart failure, diabetes mellitus and who were using hormone-replacement therapy (HRT) or tamoxifen were excluded. The outcome variable considered was patient/control status. Patients were defined as women with histologically confirmed breast cancer; controls were defined as apparently healthy, asymptomatic women. Menopausal status was defined at the moment of blood collection. A premenopausal woman was defined if she had at least one natural cycle in the previous 12 months or was younger than 48 years or after hysterectomy. A post-menopausal woman was defined if she reported a natural menopause or bilateral oophorectomy, or if she was at least 56 years. All individuals had normal thyroid-function, because the thyroid function might change after treatment for breast cancer, especially after irradiation of the purposes of logistic regression analysis, this qualitative variable was used quantitatively (patient = 1, control = 0).

All subjects were recruited from the University Hospital of Puerto Real or Misericordia Hospital (Cádiz, Spain), during the period from February 1, 2004 to November 31, 2006. All participants gave their informed consent to take part in the study, the protocol was approved by the Ethic Committee of Hospital, which was done in accordance with the recommendations of the Declaration of Helsinki.

### Clinical Parameters and Laboratory Procedures

Variables that might potentially predict disease were selected if they were known to be related with diet, and if they varied (or could be expected to vary) with tumour status: basal blood levels of glucose, insulin, HbA1c, C-peptide, IGF-I, total cholesterol, triglycerides, HDL-C, LDL-C, selenium, and BMI. All values were treated as numerical continuous variables. The prediction variables were selected by their dependency on diet and variations with tumoral status. From the initial model, including all variables, were eliminated one by one until they get the model with the best sensitivity and specificity.

Body weight was measured to the nearest 0.2 kilograms with participants dressed in lightweight clothing, and height was measured to the nearest < 0.5 centimeters after the subject shoes had been removed. BMI was calculated with the formula BMI = body weight/height^2^. Peripheral blood samples were obtained from the subjects fasted for 10-12 h before sample collection. A further 10 mL was collected in bottles and promptly transported to the laboratory at 4° C for immediate processing. Others aliquots of the sera were stored at −20°C in a storage freezer, which was not in daily use, until analysis. All determinations were carried out whitin a year and a half after sample collection.

Basal glucose and the concentration of total cholesterol and triglycerides were estimated enzymatically [23]. HDL-C was assayed enzymatically after precipitation of lipoproteins with a density of less than 1.063 g/mL by the addition of heparin and MnCl2, according to the method of Warnick [24]. LDL-C was calculated according to the Friedewald formula [23]. Basal insulin [25] and C-peptide [26] were measured with ELISA in the same sample. HbA1c was determined with the specific liquid chromatography technique described by Davis [27], IGF-I by RIA [28], and selenium by atomic absorption spectrophotometry in a graphite chamber [29].

### Statistical Analyses

Statistical analysis was performed using the Systat statistical software package [30] run on an IBM-compatible personal computer. Descriptive analyses were used for all quantitative variables. The results for the patient and control groups were compared with the Kruskal-Wallis test. Possible correlations between different variables were also sought and a logistic regression analysis was used: variables were tested and excluded until the most sensitive and specific model was obtained [31].

## RESULTS

Baseline characteristics of the cases and controls, by years, menopausal status, are shown in Table **1**. The results for pre- and postmenopausal women were analysed separately. In premenopausal women, significant differences were found between patients and control subjects in HbA1c level (p<0.001), insulin concentration (p = 0.030), C-peptide concentration (p = 0.004), IGF-I (p<0.001), total cholesterol (p<0.001) and selenium (p = 0.002) (Table **2**). Among post-menopausal women, patients with breast cancer and healthy control subjects differed significantly in basal glycaemia (p<0.001), HbA1c level (p = 0.008), C-peptide concentration (p<0.001), total cholesterol (p<0.001), HDL-C (p<0.05), LDL-C (p<0.05), selenium (p<0.001) and BMI (p = 0.101) (Table **3**).

### Logistic Regression Model for the Diagnosis of Breast Cancer in Premenopausal Women

The diet-related factors investigated here were tested by elimination to determine those which yielded an equation with maximal sensitivity and specificity. The variables included in the final equation were basal blood concentrations of glucose, insulin, C-peptide, HbA1c, IGF-I, total cholesterol, HDL-C, LDL-C, triglycerides, selenium, and BMI. The resulting linear mathematical model predicted the diagnosis with a sensitivity of 100% and a specificity of 100%. The model is the following: Diagnost = Constant + Gly X1+ Insulin X2 + C-pep X3+ HbA1c X4 + IGF-I X5 + Chol X6 + HDL-c X7 + LDL-c X8 + Try X9 + Sel X10.

The log likelihood of the constants only model was LL(0) =  −35.341 and 2*[LL(N) - LL(0)]  =  70.668; 10 d.f.; chi-squared p  =  0.000; McFadden rho-squared  =  1.000.

### Logistic Regression Model for the Diagnosis of Breast Cancer in Postmenopausal Women

The variables selected by elimination for inclusion in the linear model proposed to predict the diagnosis of breast cancer were basal blood concentrations of insulin, C-peptide, HbA1c, IGF-I, cholesterol and BMI. The resulting linear mathematical model predicted the diagnosis with a sensitivity of 100% and a specificity of 100%. The model is the following: Diagnost =  Constant + Insulin X1 + C-pep X2+ HbA1c X3 + IGF-I X4 + Chol X5 + BMI X6 . The log likelihood of the constants only model was LL(0) = −33.271 and 2∗[LL(N)-LL(0)]  =  66.533; 6 d.f.; chi-squared p = 0.000; McFadden rho-squared = 1.000.

In both cases of pre and postmenopausal women, the model coefficients are the odds ratio calculated by the Systat statistical package; but in this study they would be not significant.

## DISCUSSION

To our knowledge this is the first study to develop a linear binary logistic regression model that is highly sensitive and specific in predicting the diagnosis of breast cancer from the subject’s endocrine and metabolic profile. All the variables examined here are clearly modified by the disease and with the exception of selenium, the changes resembling those found in subjects on a hypercaloric diet. The metabolic profile differed clearly in pre- and postmenopausal women: nevertheless we identified some variables that predicted the likelihood of breast cancer in both of these groups. These differences are the reason of two separate models for pre and postmenopausal women.

Metabolic alterations were greater in postmenopausal patients, and highly significant differences between healthy control women and patients with breast cancer were found in basal concentrations of glucose, HbA1c, C-peptide, total cholesterol, selenium and in the Quetelet index. The difference between these groups approached significance for insulin, IGF-I and triglycerides concentrations, with a bigger sample size of future study, this shall be corrected.

In premenopausal women the difference in basal glucose concentration between patients and controls was not significant; in contrast, significant differences were found for gly-cosylated haemoglobin, serum insulin, C-peptide, IGF-I, total cholesterol and selenium.

Elevated glycosylated haemoglobin is a more objective measure of fluctuating glucose levels than is basal glucose concentration, which depends to some extent on the time of day when blood is withdrawn.

In postmenopausal women the difference in basal glucose levels between control subjects and patient was significant; however, the difference in insulin levels was not (p = 0.076). We draw attention to the finding that in both pre- and post-menopausal breast cancer patients, C-peptide was significantly elevated; consequently, insulin hyperproduction occurred. However, serum insulin concentrations were lower in both patient subgroups than in their respective control subgroups; the difference was significant in premenopausal women, and approached significance in postmenopausal patients.

Our study provides evidence that low serum HDL-C is independently associated with increased postmenopausal breast cancer risk among women with breast cancer. These findings suggest an interaction between metabolic disturbances (i.e., overweight or obesity and low serum HDL-C) in postmenopausal breast carcinogenesis. Probably, after menopause, bioavailable estrogens formed in adipose tissue by the aromatization of androgens are a major stimulus for breast carcinogenesis [32]. Because low HDL-C is related to increased levels of several cancer-promoting hormones (e.g., androgens, estrogens, insulin, and IGF-I), the observed association may reflect the relative importance and mutual dependence of different disease pathways in malignant breast tumours after menopause. Thus, HDL-C is a potential marker of postmenopausal breast cancer risk that could provide a means for identifying women at higher risk who may be candidates for preventive intervention in the future.

Most recently, in a large cohort of 38.823 women, low serum high density lipoprotein cholesterol, described by the authors as a part of the metabolic syndrome, was associated with increased postmenopausal breast cancer risk [33].

This finding has not been reported in any other situation; the only possible explanation is that the tumoral tissue binds more insulin than do normal tissues. Guastamacchia *et al.* reported an increase in insulin receptors in malignant tumoral tissues [10]. A comprehensive review of insulin and cancer risk is beyond the scope of this report. And our hypothesis is that this was responsible for the apparently paradoxical findings of increased insulin production and lower serum levels of insulin. Although not without controversy, the preponderance of studies suggest that insulin may influence cancers that are more commonly seen in Western population [34,35]. These authors also suggested that the increased insulin uptake in tumoral tissue might trigger mechanisms of cell growth [36,37].

Hankinson *et al.* reported an interesting finding of a positive relation between the concentration of circulating IGF-I and risk of breast cancer in premenopausal women with respect to hormone-replacement therapy in postmenopausal women [13].

The changes in metabolic and endocrine variables in patients with breast cancer simulate the pattern of changes in subjects on a carbohydrate-rich diet, which leads to more or less prolonged increase in postprandial serum glucose concentrations. This would account for the elevated basal serum concentration of glucose in postmenopausal women, and the increased Hb1Ac levels in both pre- and postmenopausal subjects. This relative hyperglycaemia increases pancreatic insulin production, as shown by the increase in C-peptide (secreted at equimolar concentrations with insulin) in both patient subgroups. Excess insulin subsequently disappears from circulation, possibly because of uptake by insulin receptors in the tumoral tissue [38]. Insulin per se is a tumoral growth factor and may stimulate other cellular growth factors [39]. The link between postmenopausal overweight women and breast cancer could be mediated through hyper-insulinemia [40].

The increase in serum total cholesterol in our pre- and postmenopausal patients also parallels the increase caused by a hypercaloric diet. In addition, postmenopausal women with breast cancer had a significantly greater BMI than their control subgroup, the difference approaching significance in premenopausal women with and without cancer. One possible explaination for the smaller difference between the latter subgroups is that women who are less overweight may have been physically more active.

Serum selenium concentration is thought to reflect dietary selenium intake. Like other authors, we found significantly lower serum levels of this mineral in patients than in controls [19]. We do not agree with (pre- and postmeno-pausal women) the current opinion on this subject and are conducting a clinical and experimental research on the subject [41].

Triglyceride levels were higher, and HDL-c, levels lower, in both pre- and postmenopausal patients than in controls; these differences were statistically significant (p<0.05). However, the postmenopausal women with cancer had significantly (p<0.05) higher LDL-c than controls.

We have proposed the hypothesis that the metabolic profile can be used to identify clinical cases of breast cancer in the general population (diagnosis). This is of particular importance for premenopausal women, for whom there is no reliable method of early diagnosis. Confirmation of a relationship of breast cancer risk hyperinsulinemia or with plasma IGF-I levels, could open up new perspectives to be used in primary prevention, as a normal reference pattern, moreover, data from the metabolic profile can be used to elaborate a mathematical model of breast carcinogenesis, or the use of certain chemopreventive drugs.

In conclusion, our findings, if confirmed, have several practical implications. Many questions remain to be answered and systematic research should be aimed at clarifying the points summarized below 1). We do not yet know whether the metabolic alterations described here precede breast cancer or appear as a consequence of this disease. Moreover, we have yet to determine whether these alterations are reversible 2). It remains to be shown whether metabolic screening and mathematical treatment of the data can detect breast cancer in a high risk population before the disease appears 3). We do not know whether our method is able to rule or diagnose breast cancer in premenopausal women when the results of mammography are inconclusive 4). The effectiveness of our approach needs to be compared with mammography, and the performance of both can be assessed against the gold standard of histological findings 5). The metabolic-endocrine profile could be used to establish normal reference values in dietetic programs of primary prevention. The lack of target values has meant that programs aimed at normalizing the metabolic profile have thus far had only limited success 6). We do not know whether the metabolic alterations induce activation oncogenes and play a role in the growth of breast cancer 7). Identifying the factors which affect these biomarkers is of particular interest as subjects at increased risk could benefit from lifestyle changes, and/or chemoprevention intervention.

## Figures and Tables

**Table 1:** Baseline Characteristics

VARIABLES	CONTROLS N=250	CASES N=204
**Premenopausal women**	125	108
Age (years)	39.6	40.3
Age at menarche (years)	12.1	12.7
**Postmenopausal women**	125	96
Age (years)	62.5	62.9
Age at menopause (years)	47.2	48.0
Age at menarche (years)	12.6	12.8

**Table 2:** Metabolic and Endocrine Parameters in a Sample of Premenopausal Women in Southern Spain

VARIABLES	CONTROLS (Means)	CI (95%)	PATIENTS (Means)	CI (95%)	P Value
**GLYCEMIE (mmol/L)**	5.04	4–6	5.50	5–5.93	= 0.294
**HbA1c (%)**	6.47	5–7	7.75	6.99–7.89	< 0.001
**INSULIN (mmol/L)**	80.93	78–82	67.01	66.30–68.01	= 0.030
**C-PEPTIDE (mmol/L)**	1.86	1.87–1.89	2.59	2.54–2.62	= 0.004
**IGF-I (kU/L)**	0.475	0.462–0.480	0.792	0.788–0.801	< 0.001
**CHOLESTEROL (mmol/L)**	4.35	4–5.02	6.07	5.09–6.32	< 0.001
**TRIGLYCERIDES (mmol/L)**	1.26	1.01–1.33	1.38	1.34–1.42	< 0.05
**HDL-C (mmol/L)**	3.56	3.42–3.61	3.51	3.48–3.55	< 0.05
**LDL-C (mmol/L)**	3.48	3.39–3.61	3.51	3.47–3.55	< 0.05
**SELENIUM (μg/L)**	92.53	90–94	80.21	79.05–81.23	= 0.002
**BMI**	25.99	24–27	27.93	26.09–28.03	= 0.115

**Table 3:** Baseline Characteristics

VARIABLES	CONTROLS(Means)	CI (95%)	PATIENTS (Means)	CI (95%)	P value
**GLYCEMIE (mmol/L)**	4.99	4.59–5.02	6.04	5.99–6.32	< 0.001
**HbA1c (mmo/L)**	6.77	6.02–6.96	7.91	7.02–8.25	= 0.008
**INSULIN (mmol/L)**	80.93	78.3–81.54	73.4	72.45–74.51	= 0.076
**C-PEPTIDE (mmol/L)**	2.06	1.99–2.56	3.54	2.99–3.69	< 0.001
**IGF-I (kU/L)**	0.525	0.500–0.563	0.71	0.66–0.75	= 0.079
**CHOLESTEROL (mmol/L)**	4.37	4.01–4.87	5.69	5.05–6.31	< 0.001
**TRIGLYCERIDES (mmol/L)**	1.31	1.29–1.32	1.65	1.60–1.72	< 0.05
**HDL-C (mmol/L)**	1.60	1.56–1.68	1.48	1.46–1.52	< 0.05
**LDL-C (mmol/L)**	3.34	3.30–4.1	4.03	3.99–4.56	> 0.05
**SELENIUM (μg/L)**	89.79	88.63–90.3	75.16	74.69–75.99	< 0.001
**BMI**	25.61	24–26	26.85	24.36–27.12	= 0.010
